# Clavicle Shaft Non-Unions–Do We Even Need Bone Grafts?

**DOI:** 10.3390/jcm13164850

**Published:** 2024-08-16

**Authors:** Nils Mühlenfeld, Ferdinand C. Wagner, Andreas Hupperich, Lukas Heykendorf, Andreas Frodl, Peter Obid, Jan Kühle, Hagen Schmal, Benjamin Erdle, Martin Jaeger

**Affiliations:** 1 Department of Orthopedics and Trauma Surgery, Medical Centre-Albert-Ludwigs-University of Freiburg, Faculty of Medicine, Albert-Ludwigs-University of Freiburg, 79106 Freiburg, Germany; ferdinand.wagner@uniklinik-freiburg.de (F.C.W.); andreas.hupperich@uniklinik-freiburg.de (A.H.); lukas.heykendorf@uniklinik-freiburg.de (L.H.); andreas.frodl@uniklinik-freiburg.de (A.F.); peter.obid@uniklinik-freiburg.de (P.O.); jan.kuehle@uniklinik-freiburg.de (J.K.); hagen.schmal@uniklinik-freiburg.de (H.S.); benjamin.erdle@uniklinik-freiburg.de (B.E.); martin.jaeger@uniklinik-freiburg.de (M.J.); 2 Department of Orthopedic Surgery, University Hospital Odense, Sdr. Boulevard 29, 5000 Odense, Denmark

**Keywords:** clavicle fracture, pseudarthrosis, shoulder surgery, revision, bone graft, healing rate, relative stability

## Abstract

**Background:** The surgical treatment of bony non-unions is traditionally performed with additional bone grafts when atrophic and/or stronger implants when hypertrophic. In the case of the clavicle shaft, however, in our experience, a more controversial method where no additional bone graft is needed leads to equally good consolidation rates, independent of the non-union morphology. This method requires the meticulous anatomical reconstruction of the initial fracture and fixation according to the AO principle of relative stability. **Methods:** A retrospective review following the STROBE guidelines was performed on a consecutive cohort of all patients who received surgical treatment of a midshaft clavicle non-union at the Medical Center of the University of Freiburg between January 2003 and December 2023. Patients were identified using a retrospective systematical query in the Hospital Information System (HIS) using the International Statistical Classification of Diseases and Related Health Problems Version 10 (ICD-10) codes of the German Diagnosis Related Groups (G-DRG). Two groups were formed to compare the consolidation rates of patients who received additional bone grafting from the iliac crest with those of patients who did not. A 3.5 mm reconstruction LCP plate was used in all patients. Consolidation rates were evaluated using follow-up radiographs and outcomes after material removal with a mean follow-up of 31.5 ± 44.3 months (range 0–196). **Results:** Final data included 50 patients, predominantly male (29:21); age: 46.0 ± 13.0 years, BMI 26.1 ± 3.7. Autologous bone grafts from the iliac crest were used in 38.0% (*n* = 19), while no bone addition was used in 62.0% (*n* = 30). Six patients were lost to follow-up. Radiological consolidation was documented after a mean of 15.1 ± 8.0 months for the remaining 44 patients. Consolidation rates were 94.4% (*n* = 17) in patients for whom additional bone grafting was used and 96.2% (*n* = 25) in patients for whom no graft was used. There was no relevant difference in the percentage of atrophic or hypertrophic non-unions between both groups (*p* = 0.2425). Differences between groups in the rate of consolidation were not significant (*p* = 0.7890). The complication rate was low, with 4.5% (*n* = 2). **Conclusions:** Independent of the non-union morphology, non-unions of the clavicle midshaft can be treated successfully with 3.5 mm locking reconstruction plates without the use of additional bone grafting in most cases.

## 1. Introduction

Non-unions of the clavicle are rare and have been documented in between 0.1 and 24% of cases following mostly non-operative treatment [1,2,3,4]. Early research identified contributing factors such as clavicle shortening, female sex, fracture comminution, and displacement as well as older patient age [5]. In midshaft fractures, depending on the study, rates of suffering a non-union after non-operative treatment have been reported as 4.5–6.2% within 24 weeks after trauma [6,7,8]. Patients with non-unions have inferior outcomes compared to patients with adequate fracture healing with mostly pain but also shortening, limited range of motion, and strength deficits [9].

**Figure 1:**
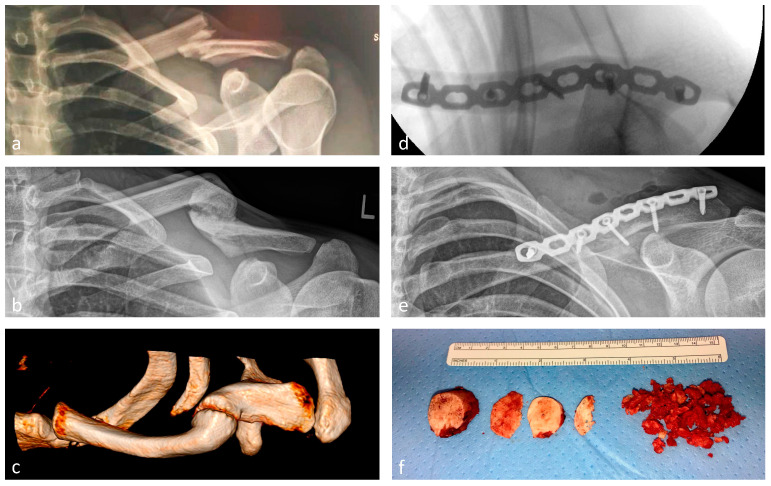
(**a**) Anterior–posterior radiograph of a fracture of a left clavicle shaft; (**b**) anterior–posterior radiograph of the developed symptomatic hypertrophic non-union after non-operative treatment; (**c**) 3D reconstruction via computer tomography; (**d**) intraoperative radiograph in axial direction after the resection of the non-union and internal fixation with an anatomically bent and twisted 3.5 mm LCP reconstruction plate combined with an interfragmentary lag screw; (**e**) postoperative anterior–posterior radiograph to demonstrate the adequate length of the clavicle and the anterior–superior placement of the plate osteosynthesis; (**f**) intraoperative picture of the resected hypertrophic non-union.

Th surgical treatment of clavicle fractures can result in atrophic as well as hypertrophic non-unions if the biomechanics of the given fracture are not addressed correctly. Revision surgery is needed in both atrophic and hypertrophic symptomatic non-unions. The often multifactorial pathophysiology has to be identified and correctly addressed to treat non-unions successfully [10]. The revision surgery of clavicle shaft non-unions is traditionally performed by the resection of the non-union area and plate (re)osteosynthesis with the addition of cancellous bone or a tricortical bone graft from the iliac crest to either enhance vital biology locally and/or restore the original length. Depending on the underlying pathophysiology, different approaches are recommended in the literature [10]. Evidence-based treatment guidelines do not yet exist due to the lack of data with high case numbers of follow-ups after surgically treated non-unions. There are, however, certain commonly accepted rules concerning the treatment of bony non-unions. In the case of hypertrophic non-unions, for example, most authors recommend the use of a more rigid plate or even the use of double plating to form a more stable construct and enable consolidation after bone graft insertion [11]. Disadvantages in the use of bone grafts, however, are increased operative time and blood loss, as well as a considerably high rate of donor-site morbidity [12,13,14]. Having an evidence-based understanding of non-union treatment in mind, we tried to optimize these techniques to avoid any bone grafting and therefore avoid any donor-side morbidity. The main aspects are meticulous soft tissue handling, preserving the soft tissue, and intrinsic blood supply as well as possible. In addition, a thorough identification and cleaning of all remaining fragments is performed, allowing for an anatomic restoration of the clavicle and the opening of any sclerosis to the main fragments, followed by a semi-rigid, long-spanning plate osteosynthesis using a 3.5 mm reco-LCP. This plate is bent and twisted to fit anatomically, pulled to the bone by eccentrical cortical screws, and fixed with angle-locking screws. It is combined with a preceding interfragmentary lag screw if possible and, if existent, preserved local callus placed around the fracture site (Figure 1). This method has been intermittently performed at our level-I-trauma center over the past few decades, independent from initial fracture type or non-union characteristics.

This retrospective study aimed to evaluate the consolidation rate after the surgical treatment of clavicle shaft non-unions with the use of bone grafts harvested from the iliac crest in comparison to those without. We hypothesized that there is no significant difference in the rate of consolidation between both groups, and it is therefore possible to avoid bone grafting in the treatment of symptomatic clavicle shaft non-unions in most cases.

## 2. Materials and Methods

Approval from the institutional review board was obtained before performing this retrospective study (24-1046-S1-retro). This study followed the STROBE guidelines for observational studies (Strengthening the Reporting of Observational Studies in Epidemiology) and the RECORD guidelines (Reporting of studies Conducted using Observational Routinely Collected Data) [15,16].

A retrospective review was performed on a consecutive cohort of all patients with a midshaft clavicle non-union at the authors’ institution between January 2003 and December 2023. Patients were identified via a retrospective query of the hospital’s electronic medical records using the International Classification of Diseases Version 10 (ICD-10) codes of the German Diagnosis Related Groups (G-DRG). Patients’ characteristics, disease-specific information, radiologic characteristics of the mal-unions, type of surgical management, and outcomes were abstracted and transferred to an electronic spreadsheet.

Patients ≥18 years of age with a documented non-union of the clavicle midshaft who received operative treatment were eligible for inclusion. Patients with asymptomatic stable non-unions that remained non-operative were excluded from the dataset. Patients underwent standard radiological diagnosis including radiographs of the clavicle and in some cases computer tomography (CT). Patients without any radiological control later than six weeks postoperatively were defined as “lost to follow-up” and excluded from the final evaluation of consolidation rates. Non-unions were classified as symptomatic non-unions with concomitant symptoms such as pain, limited range of motion, or numbness. The initial management of the clavicle fracture was performed either in the author’s institution or a different hospital. All patients were treated by specialized orthopedic trauma surgeons. The following two groups were compared in this study:

“Graft”: Patients who underwent the implantation of autologous cancellous bone and/or a tricortical bone graft from the iliac crest.

“No Graft”: Patients who received surgical treatment of the non-union without the additional use of bone grafts harvested from the iliac crest.

After surgery, no specific immobilization was applied or recommended. Aftercare was limited to a recommendation of avoiding weightlifting for 6 weeks postoperatively. In cases of non-unions suspected to be caused by bacterial infection, tissue samples were taken intraoperatively for microbiological analysis, and antibiotic therapy was initiated accordingly.

### Statistical Analysis

All variables were evaluated for the distribution of normality using a combination of histograms, quantile–quantile (Q–Q) plots, and Shapiro–Wilk tests. Descriptive statistics were summarized as means and standard deviations for quantitative variables and as counts and frequencies for categorical variables. The significance of mean differences between continuous variables had a normal distribution. Differences between consolidation rates in the treatment groups were compared using the chi-squared test and Fisher’s exact test. Statistical significance for all comparisons was set at *p*  <  0.05. All analyses were performed with Stata statistical software version 10.0 (GraphPad Prism).

## 3. Results

### 3.1. Sociodemographic Data

Of the 60 patients with a coded non-union of the clavicle shaft, 7 were excluded from the dataset as documentation was inconclusive or the non-union was not evident (incorrect coding/documentation). In three patients, the non-union proved to be stable and asymptomatic after the removal of the initial stabilization material. Hence, no reosteosynthesis was necessary, and the datasets were excluded (no revision surgery of non-union). The final data included 50 patients who presented with symptomatic non-unions for (revision) surgery at our level-I-trauma center. Of these, 21 were female (42.0%), and 29 were male (58.0%); the mean age was 46.0 ± 13.0 years (range 21–68 years), the mean BMI was 26.1 ± 3.7 (range 18–33), and 34.0% (*n* = 18) of patients were active nicotine smokers. Patients underwent follow-ups within a mean period of 31.5 ± 44.3 months postoperatively (range 0–196). Table 1 presents additional information and the results for each group of patients individually.

### 3.2. Characteristics of Non-Union

The leading symptom in all patients (*n* = 50) was persisting or aggravating pain. Most of the fractures leading to non-unions had been initially treated non-operatively (64.0%, *n* = 32), whereas only 36.0% (*n* = 18) had been treated operatively. Of the latter, 66.6% (*n* = 12/18) had initially been treated via plate osteosynthesis and 33.3% (*n* = 6/18) via intramedullary nail (TEN). All but one of the operatively treated patients had undergone surgery primarily at other hospitals before presenting at our department for the treatment of their symptomatic non-union. Non-unions were classified as atrophic in 58.0% (*n* = 29) and hypertrophic in 40.0% (*n* = 20) of the cases. There was no relevant difference (*p* = 0.5514) in the rate of atrophic and hypertrophic non-unions between the initially operatively treated and non-operatively treated patients. Additionally, there was no relevant difference in the percentage of atrophic vs. hypertrophic non-unions between both groups (*p* = 0.2425) as in “Graft Patients”, 13 non-unions were labeled as atrophic and 6 hypertrophic, while in the “No Graft Patients”, 16 non-unions were labeled atrophic and 15 hypertrophic. Non-union characteristics did not influence consolidation rates, as radiological consolidation was documented for 17/17 hypertrophic and 25/27 atrophic non-unions (100% vs. 92.6%, *p* = 0.515). Only in one case could a bacterial infection be identified as the cause for non-union after primary fracture plate osteosynthesis. The infection was diagnosed via intraoperative tissue sampling and culturing, with Proteus mirabilis being the identified cause.

### 3.3. Treatment of Clavicle Non-Unions and Consolidation Rates

**Figure 2:**
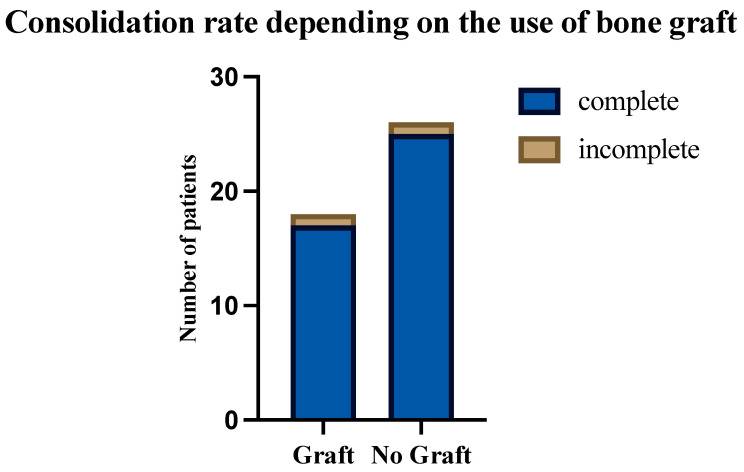
Number of patients who reached complete or incomplete radiological consolidation in comparison between surgical methods (*p* = 0.7890).

Of the whole cohort, six patients could not be included in the final evaluation of consolidation rates because they did not show up for further follow-up appointments, and no further postoperative radiological images were available (“lost to follow-up”). In all of the remaining 44 patients, a plate osteosynthesis with a 3.5 mm reconstruction locking plate was performed after the vitalization/resection of the atrophic or hypertrophic area of the clavicle non-union. In 40.9% (*n* = 18), additional bone graft, harvested from the iliac crest, was used (Graft), while 59.1% (*n* = 26) received no additional bone grafting (No Graft). In 95.5% (*n* = 42) of patients, radiological consolidation was documented after a mean period of 15.1 ± 8.0 months (range: 4–39). Rates of radiological consolidation between the two different treatment modalities were not significantly different (*p* = 0.7890) as radiological consolidation rates were 96.2% (*n* =25) for “No Graft Patients” and 94.4% for “Graft Patients” (*n* = 17) (Figure 2).

The implanted osteosynthesis material was removed in 74.0% (*n* = 37) of the patients after a mean of 18.2 ± 8.1 months (range: 6–41), with impeding hardware being the predominant reason. Table 2 demonstrates the outcome data for each group individually.

### 3.4. Complications

The complication rate in the forty-four patients with follow-up data was low, with non-successful non-union treatment occurring in only two cases (4.5%), one of the “Graft Patients” and one of the “No Graft Patients”. Initial treatment prior to non-union surgery had been non-operative in both patients. In one patient, a refracture occurred with the failure of material. The first patient underwent revision surgery with reosteosynthesis after the debridement of the renewed non-union, which was successful as the material could be removed 17 months after revision surgery. The second patient refused further revision surgery and presented with persisting pain in the last follow-up after 30 months after which he did not reappear for further follow-up. There was no postoperative infection documented in the data of this study. Overall, the success of our surgical protocol was documented for 95.5% (42/44) of all patients, which can be considered excellent independent of the use of a bone graft.

## 4. Discussion

The most important finding of this study is that, independent of the morphology, non-unions of the midshaft of the clavicle can be treated successfully with 3.5 mm locking reconstruction plates, without the necessity to use additional bone graft from the iliac crest. The results from this study presented no relevant difference in the rate of bone union compared to patients who did not receive additional bone transfer from the iliac crest or were treated with the addition of a tricortical iliac crest bone. Therefore, graft augmentation did not influence the rate of bony union, and its necessity can be discussed. Donor site comorbidities like pain, wound healing disorders, or even fractures can be completely avoided in the future, respecting these findings.

Traditionally, depending on the morphology of the non-union, the underlying and often multifactorial pathology needs to be analyzed to restore the missing factors [10]. Ground principles for the treatment of the non-union like mechanical stability through correct material selection should be met. It is usually proclaimed that in cases with larger bone defects, additional bone grafting should be performed with the insertion of a tricortical iliac crest bone and cancellous bone from the iliac crest in the defect area to reach the correct length of the clavicle and good stability [17,18,19]. Additionally, in hypertrophic non-unions, in particular, it is further recommended to use more rigid osteosynthesis for clavicle non-union revision, sometimes even with the use of two stable plates as the underlying pathology is the lack of stability [20]. Atrophic non-unions are known to be caused by the inadequate angiogenesis of the defect gap after initial trauma or the removal of too much soft tissue in the initial surgical treatment, which is believed to cause osteonecrosis [10,20]. In operatively treated diaphyseal fractures, insufficient reduction and fixation additionally increase the risk for a non-union significantly [21]. Next to the presence of growth factors and osteogenic cells, an osteoconductive scaffold and mechanical stability are further essential factors to support fracture healing [22]. The use of drilling holes with a K-Wire should be considered to promote the transfer of mesenchymal stem cells (MSCs) into the intramedullary space, which is analog to the reaming procedure in larger bone shaft non-unions [21].

**Figure 3:**
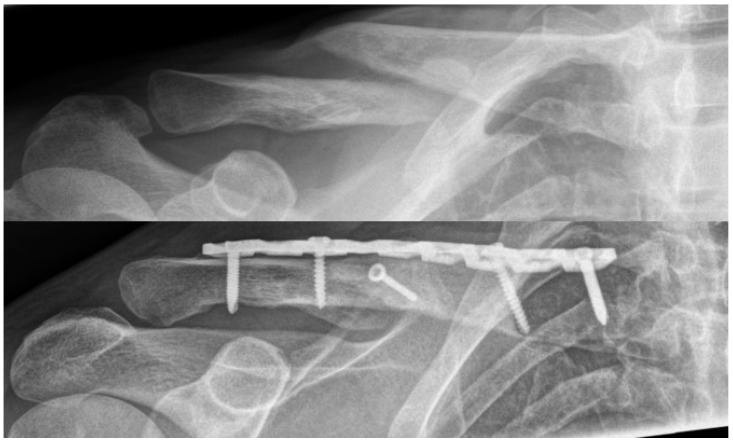
Examples of anterior–posterior radiographs of an atrophic nonunion following non-surgical treatment and following revision surgery without bone graft.

Data from this study suggest, however, that it seems to be possible—at least at the clavicle—to avoid bone grafting in the treatment of symptomatic non-unions, independent of the nature of non-union and thereby unifying their treatment. This concept certainly excludes cases with defects or relevant shortening, which require restoration by structural bone grafting. The key factors of such an approach are meticulous soft tissue handling and preserving the soft tissue and intrinsic blood supply as well as possible. In addition, a thorough identification and cleaning of all remaining fragments allowing for an anatomic restoration of the clavicle is crucial. All newly formed tissue must be removed. Sclerosis to the main fragments should be opened with a drill of K-Wire. Both direct and indirect healing principles can be applied successfully, using a semi-rigid, long-spanning plate osteosynthesis using a 3.5 mm reco-LCP. If existent, preserved local callus can be placed around the fracture site (Figure 3).

The treatment of non-unions without using autologous bone grafting has also been reported to be successful by Chen et al. (2018), who found good values in the DASH and Constant–Murley Score in a case series of 17 patients [9], as well as by Wiss and Garlich, who found no relevant difference in the healing rate for the use of bone grafting versus no grafting in a recent case series of 71 patients [17].

If performed correctly, the removal of the plate is a safe procedure that can improve outcomes like range of motion and patient discomfort [23]. In this study, no refractures after plate removal were documented. In addition, no difference in the bony consolidation between etiologies of shaft non-unions could be noted. Interestingly, we found graft-augmented osteosynthesis to be removed significantly earlier than osteosynthesis for those without iliac bone graft, although time to radiological consolidation was not significantly prolonged. A prospective radiological and clinical study could evaluate whether iliac bone augmentation possibly increases patients’ complaints and urge for implant removal.

Mills et al. reported entirely unexpected infections in 5% of their patients and reported an infection rate of non-unions in up to 38% of the cases [20]. This does not seem to be evident for non-unions of the clavicle shaft as infection was the identified cause in only a single case in this study.

Overall, depending on the scientific source, the non-unions of the clavicle are still labeled as rare occurrences, ranging from 0.1% to 15% within 24 months after trauma. The risk varies depending on the location of the fracture [7,8,24]. Multiple studies attempted to investigate the contributing factors to the development of a clavicle non-union. In this context, a displacement over one shaft width and the shortening of fracture fragments, comminution, refracture, open fractures, polytrauma, and inadequate mobilization have been mentioned [24,25]. Additional predictive epidemiological factors such as female sex and older patient age, as well as osteoporosis, might increase the risk of non-union after a clavicle fracture [22,25,26,27]. We could demonstrate a predominantly male as well as an adult cohort with non-unions of the clavicle shaft; however, as only 4.0% of the patients were over 65 years of age, only 34.0% were active nicotine smokers, and consolidation rates were high, independent of BMI. With the correct surgical method, a high consolidation rate can therefore be achieved independent of age and BMI, as demonstrated in this study, which is in line with recent studies examining the treatment of non-unions in elderly patients in the femur and humerus [28].

Because of the low incidence of a clavicle non-union, however, surgeons with sufficient experience in treating them are rare, and rates of revision surgery remain low, especially in smaller hospitals. Nevertheless, in rare case injuries, the quality of the outcome increases with the number of cases treated [29]. This can be confirmed with data from this study, as the authors of this study could demonstrate excellent outcomes in a level-I trauma center, which treats around 2-3 non-unions of the midshaft annually. If not treated correctly, persistent non-unions and refractures after plate removal are feared and frequently lead to complications [30,31,32], which only occurred in two patients in this study.

### Limitations

This study has several limitations, starting with its retrospective design and the fact that it is a report of a large case series without any comparison between treatment modalities, as all patients underwent surgical intervention for their clavicular non-union. Additionally, no clinical outcomes were evaluated. However, a larger number of cases with complete fracture consolidation throughout the documented period could be presented. Data from this study did not allow for a retrospective evaluation of decision making for the use of graft or no graft. Due to the lack of randomization in this retrospective study, there is a possible risk of treatment selection concerning different characteristics that were not controlled for in patients who did or did not receive a graft.

## 5. Conclusions

Independent of the non-union morphology, non-unions of the clavicle midshaft can be treated successfully with 3.5 mm locking reconstruction plates without the use of additional iliac bone grafting in most cases. Further investigation in terms of prospective multicenter studies is mandatory to provide a guideline basis for these rare but challenging cases.

## Figures and Tables

**Table 1:** Demographic data of the whole cohort for each patient group individually; values are reported in either n (%) or mean ± SD.

	Graft	No Graft
Number of Patients	19	31
Age (years)	46 ± 13.4	45 ± 12.7
Sex		
- Female	8 (42.1)	13 (61.3)
- Male	11 (57.9)	18 (58.1)
BMI	27.8 ± 3.8	25.0 ± 3.2
Nicotine	7 (36.8)	10 (32.3)
Non-union type		
- Atrophic	12 (63.2)	16 (51.6)
- Hypertrophic	5 (63.2)	15 (48.4)
- Infect	1 (5.3)	0 (0.0)
Initial management		
- Surgical	7 (36.8)	10 (32.3)
- Non-surgical	12 (63.2)	21 (67.7)

**Table 2:** Outcome data for each patient group individually; values are reported in either *n* (%) or mean ± SD.

	Graft	No Graft	*p*
Radiological consolidation rate	17/18 (94.4)	25/26 (96.2)	0.7890
Time to consolidation (months)	13.8 ± 6.0	16.0 ± 9.1	0.3744
Plate removal	15/18 (83.3)	22/26 (84.6)	1.0000
Time to plate removal (months)	14.8 ± 5.7	20.6 ± 8.6	0.0163 *
Lost to follow-up (* regarding whole cohort)	1 (5.6)	5 (16.1)	0.3873

* Statistically significant.

## Data Availability

Data are unavailable for public view due to privacy or ethical restrictions.
